# Kidney Stone Prevention

**DOI:** 10.1016/j.advnut.2023.03.002

**Published:** 2023-03-09

**Authors:** Paleerath Peerapen, Visith Thongboonkerd

**Affiliations:** Medical Proteomics Unit, Research Department, Faculty of Medicine Siriraj Hospital, Mahidol University, Bangkok, Thailand

**Keywords:** bioactive compound, citrate, diuresis, natural compound, nephrolithiasis, probiotics, protection, urolithiasis

## Abstract

Kidney stone disease (KSD) (alternatively nephrolithiasis or urolithiasis) is a global health care problem that affects people in almost all of developed and developing countries. Its prevalence has been continuously increasing with a high recurrence rate after stone removal. Although effective therapeutic modalities are available, preventive strategies for both new and recurrent stones are required to reduce physical and financial burdens of KSD. To prevent kidney stone formation, its etiology and risk factors should be first considered. Low urine output and dehydration are the common risks of all stone types, whereas hypercalciuria, hyperoxaluria, and hypocitraturia are the major risks of calcium stones. In this article, up-to-date knowledge on strategies (nutrition-based mainly) to prevent KSD is provided. Important roles of fluid intake (2.5–3.0 L/d), diuresis (>2.0–2.5 L/d), lifestyle and habit modifications (for example, maintain normal body mass index, fluid compensation for working in high-temperature environment, and avoid cigarette smoking), and dietary management [for example, sufficient calcium at 1000–1200 mg/d, limit sodium at 2 or 3–5 g/d of sodium chloride (NaCl), limit oxalate-rich foods, avoid vitamin C and vitamin D supplements, limit animal proteins to 0.8–1.0 g/kg body weight/d but increase plant proteins in patients with calcium and uric acid stone and those with hyperuricosuria, increase proportion of citrus fruits, and consider lime powder supplementation] are summarized. Moreover, uses of natural bioactive products (for example, caffeine, epigallocatechin gallate, and diosmin), medications (for example, thiazides, alkaline citrate, other alkalinizing agents, and allopurinol), bacterial eradication, and probiotics are also discussed.

**Statement of Significance**

This review provides comprehensive and up-to-date knowledge on strategies to prevent KSD. These include increased fluid intake and diuresis, lifestyle and habit modifications, dietary management, and uses of natural bioactive products, medications, bacterial eradiation, and probiotics.

## Introduction

Deposition of inorganic substances (such as crystalline salts) together with organic components (such as urinary macromolecules) inside renal parenchyma or pelvicalyceal system leads to kidney stone formation. Kidney stone disease (KSD) (alternatively nephrolithiasis or urolithiasis) is common in almost all areas of the world, with continuously increasing prevalence in several regions [[1], [2], [3], [4]]. Recently, data analyses using the information from the NHANES have revealed the higher KSD prevalence in males than in females among US adult population [5]. However, its prevalence in men has been stable during the last decade (11.6% during 2007–2008 and 11.9% during 2017–2018) but has risen in women (from 6.5% during 2007–2008 to 9.4% during 2017–2018) [5]. In addition, KSD occurs in a wide range of ages, including children, adolescents, and adults [1,5].

Currently, there are a variety of effective therapies available for KSD. The most commonly used methods are surgical removal strategies, such as extracorporeal shock wave lithotripsy, ureteroscopy, and percutaneous nephrolithotomy. The method of choice for stone removal depends largely on size and location of the stone. Nevertheless, recurrence of the stone after removal remains a big problem for surgical management of KSD. A recent report from Iceland has shown that recurrence rate among children after the stone removal ranges from 26% at 5 y postsurgery to 46% at 20 y postsurgery [6]. Because the cost for stone removal is considerably high, decreasing numbers of patients with new and recurrent KSD (stone formers) would reduce the overall cost of KSD management. In addition to financial burden, KSD causes substantial physical and mental burdens to the stone formers [7]. Therefore, the preventive strategies for new and recurrent stone formation should be more seriously considered.

With multistep processes, stone formation is influenced by several intrinsic factors (for example, genetic background) and extrinsic factors (for example, diets, behaviors, and infections) [8]. Reducing the etiologies and/or risk factors for KSD is supposed to be the most effective way to prevent this disease. To date, several suggestions or guidelines have been proposed for KSD prevention [[9], [10], [11], [12]]. Although most of the recommendations are consistent among these various suggestions and guidelines, some of them differ. Thus, this review summarizes and updates the knowledge on the current strategies for KSD prevention focusing on studies during the past 10 y with special emphasis on calcium stones, which is the most common among all kidney stone types. It is not our intention to devalue or ignore the works by authoritative experts (for example, Smith, Pak, Coe, Robertson, Siener, Rodgers, Tiselius, and their colleagues) in kidney stone research performed before the mentioned timeframe. Indeed, their previous works had laid the basis of the more recent studies reported in this review. Note that their original works had been frequently cited in many other previous reviews. Herein, we select to focus on the recent progress in this field.

## Type of Kidney Stones and Their Risk Factors

### Calcium stone

**FIGURE 1:**
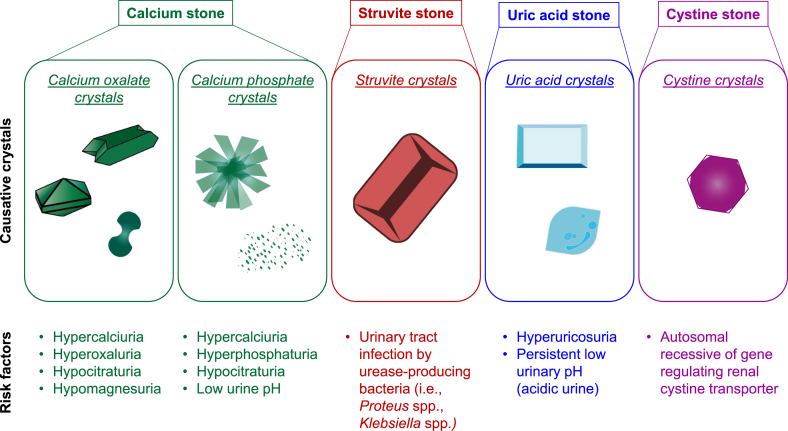
Type of kidney stones and their risk factors. Kidney stones are generally classified based on their main crystalline composition. Each of these stone types has similar and unique risk factors.

Generally, kidney stones are classified based on their main crystalline composition (Figure 1). Several studies from different regions have consistently reported that the most common inorganic composition among all kidney stones is calcium [13,14]. Calcium stone is most frequently made of calcium oxalate (CaOx), either homogeneously or mixed with others, such as calcium phosphate (CaP) [13,14]. CaOx has 3 crystalline forms based on its hydration status. These include CaOx monohydrate (COM; CaC_2_O_4_·H_2_O), CaOx dihydrate (COD; CaC_2_O_4_·2H_2_O), and CaOx trihydrate (CaC_2_O_4_·3H_2_O). Among them, COM (also called whewellite) is the most common hydrate form found in clinical stones followed by COD (also called weddellite) [13]. Hypercalciuria, hyperoxaluria, hypocitraturia, and hypomagnesuria have been recognized as the major risks of calcium stones. In particular, hyperoxaluria favors urinary COM crystallization, whereas hypercalciuria favors urinary COD crystallization [14]. CaP in the form of apatite, either hydroxyapatite [Ca_5_(PO_4_)_3_OH] or carbapatite (carbonated apatite) [Ca_10_(PO_4_)_6_CO_3_], is more common than brushite (CaHPO_4_·2H_2_O) [13,14]. Pure CaP stone is rarely seen because it usually mixes with other crystals, especially CaOx. Both CaOx and CaP have some common metabolic risk factors, such as hypercalciuria and hypocitraturia. However, CaP crystal is more susceptible to the urine pH [14].

### Struvite stone

Struvite stone comprises magnesium ammonium phosphate (MgNH_4_PO_4_·6H_2_O) and is commonly referred to as infection stone. Struvite commonly combines with CaOx and CaP, especially carbapatite, in the stone matrix [15]. This type of the stone is associated with urinary tract infection (UTI) by urease-producing bacteria, such as *Proteus* spp. and *Klebsiella* spp. [16]. Such infection leads to increased production of ammonium that causes urinary alkalinization, thereby facilitating the formation of struvite crystals [16]. As expected, a recent study has demonstrated that the stone formers with high-struvite composition in kidney stone matrix are associated with positive urine culture for *Proteus* spp. [17]. In addition to urease-producing bacteria, others (that is, *Escherichia coli* and *Enterococcus* spp.) are also found to be associated with struvite stone [16,17]. Interestingly, this stone type is more common in females than in males in some regions [18].

### Uric acid stone

Uric acid stone comprises uric acid crystals that are normally crystallized in acidic urine, and most of the uric acid crystals are in the dihydrate form [14]. This stone type is particularly common in patients with type 2 diabetes and obesity. The prevalence of uric acid stone seems to be increasing in males over recent years [18]. However, uric acid crystals mostly mix with other types of crystals [18]. Hyperuricosuria and persistent or overly low urinary pH (acidic urine) are the major risk factors for uric acid stone formation.

### Cystine stone

Cystine stone is a rare kidney stone type associated with cystinuria, a genetic disorder. It is caused by autosomal recessive gene, such as *SLC3A1*, which regulates renal cystine transporter [19]. This genetic disorder results in defective cystine transport, leading to a decrease in urinary cystine reabsorption and subsequently increased urinary cystine concentration or cystinuria [20]. Under the normal urine pH (below 6.5), cystine is relatively insoluble in the urine, leading to its precipitation, crystallization, and formation of cystine stone [19,20].

### Brief Mechanisms of Kidney Stone Formation

**FIGURE 2:**
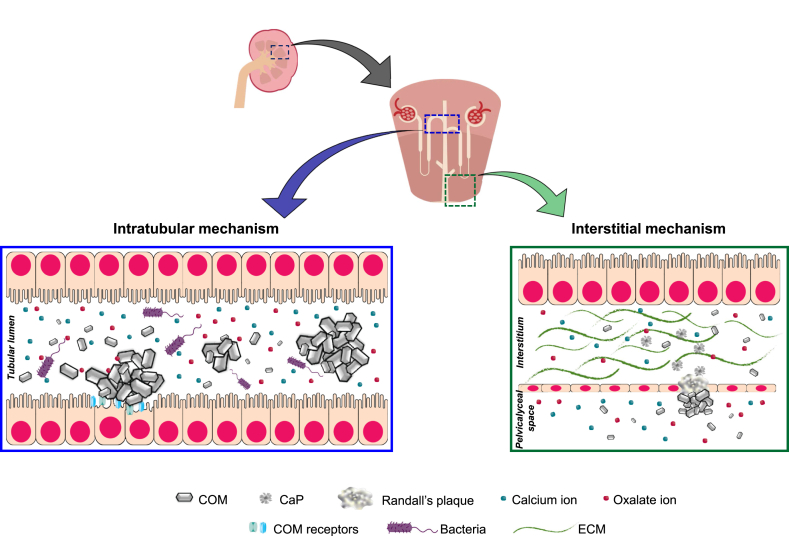
Mechanisms of CaOx stone formation. The first mechanism takes place within tubular lumens involving supersaturation of crystalline salts, crystallization, growth, self-aggregation, and adherence on tubular epithelial cells. Bacteria (both urease-producing and non–urease-producing groups) also play roles in this intratubular mechanism. The second mechanism initially takes place at renal interstitium by forming the so-called Randall plaque, which is a result of interstitial hydroxyapatite CaP crystal deposition and tissue inflammation. Some of the Randall plaques at and adjacent to the papillary tip can erode into the pelvicalyceal system, where CaOx is commonly supersaturated and crystallized. CaOx crystals subsequently deposit on the eroded Randall plaque, which then serves as the stone nidus, and the stone starts to form. CaOx, calcium oxalate; CaP, calcium phosphate; COM, calcium oxalate monohydrate; ECM, extracellular matrix.

Formation of CaOx stones, which is the most common type, can occur through 2 main mechanisms, based on its primary location (Figure 2). The first mechanism originates within tubular lumens (namely intratubular mechanism), whereas the second mechanism primarily occurs within interstitial space (namely interstitial mechanism). The first mechanism starts with supersaturation of crystalline salts, followed by crystallization within renal tubular lumens [8]. Then, the crystals are retained inside tubular lumens by crystal growth, aggregation, and adherence on apical side of tubular epithelial cells [21]. Crystal adherence on the membrane is facilitated by an increased surface expression of certain crystal-binding proteins [21]. Several studies have reported the increased surface expression of crystal-binding proteins due to stimuli, such as calcium-induced overexpression of annexin A1, oxalate-induced overexpression of α-enolase, and uric acid-induced overexpression of heat shock protein 90 [22]. The adhered crystals can further grow and self-aggregate until they cannot pass through the tubular lumen, thereby accumulating inside the renal tubules and obstructing the tubular fluid flow [8].

The second mechanism initially takes place at renal interstitium by forming the so-called Randall plaque, which is a result of interstitial hydroxyapatite CaP crystal deposition and tissue inflammation [23]. Within the microenvironment favoring supersaturation, CaP commonly crystallizes mainly at the basement membrane of the thin limb of Henle loop in the renal papillary interstitium [23]. Accumulation of interstitial CaP crystals then triggers inflammatory processes, leading to Randall plaque formation. At this location (mainly at and adjacent to the papillary tip), some of these Randall plaques can erode into the pelvicalyceal system where CaOx is commonly concentrated and crystallized [8,23]. CaOx crystals subsequently deposit on the eroded Randall plaque, which then serves as the stone nidus, and the stone starts to form [8,23].

## Strategies to Prevent KSD

### Search strategy and selection criteria

**FIGURE 3:**
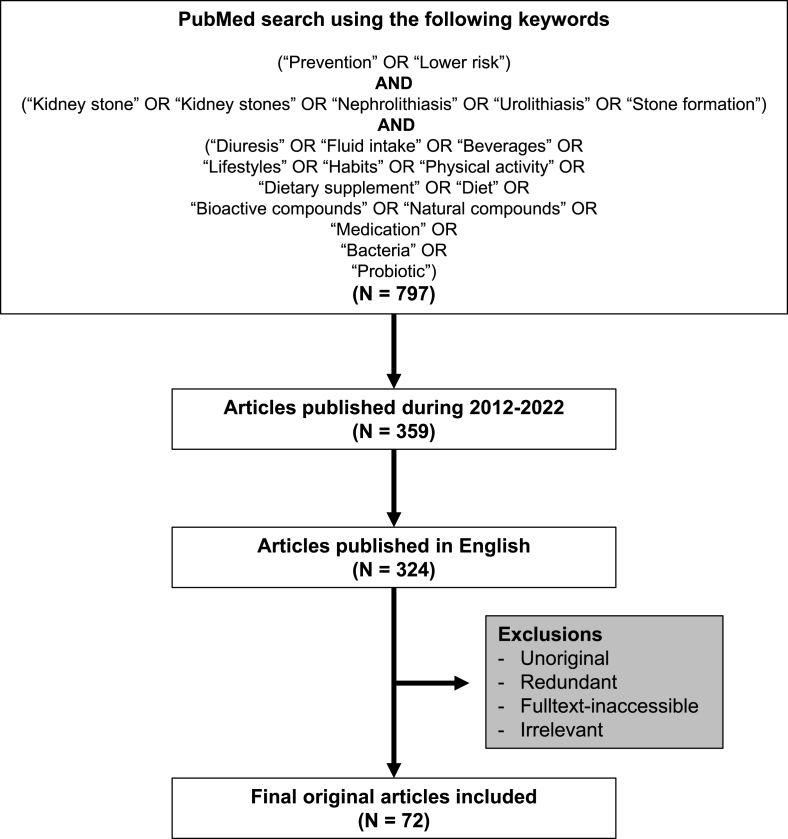
Search parameters and criteria. The schematic illustration summarizes all keywords used for PubMed search and criteria to retrieve articles for discussion in this review.

The data used for this section were mainly from previously published articles retrieved from PubMed search using the search parameters and criteria as indicated in Figure 3. Only the original articles published in English during 2012–2022 were included, whereas the redundant and full-text–inaccessible articles were excluded. In addition, the articles that did not meet such search parameters and criteria but were related to and essential for the contents in this section [for example, the latest guidelines from the American Urological Association (AUA) [9], European Association of Urology (EAU) [10], Canadian Urological Association (CUA) [11], and Urological Association of Asia (UAA) [12]] were included for a more complete discussion.

### Increased fluid intake and diuresis

Dehydration is recognized as a general risk of KSD. Increased fluid intake to maintain hydration status and to reduce urinary concentration is a long-standing and well-recognized recommendation for KSD prevention. Diuresis is a process to increase urinary volume that can be achieved by high fluid intake and using diuretic agents. The increased urine flow rate and/or reduced water reabsorption by diuretic process then contribute to increased urine volume and decreased urine osmolarity. Note that the AUA, EAU, CUA, and UAA consistently recommend to maintain the urine output >2.0–2.5 L/d with a fluid intake at 2.5–3.0 L/d [[9], [10], [11], [12]]. A clinical trial has demonstrated the association between high urine volume and lower risk of recurrent calcium nephrolithiasis [24]. Therefore, the large-volume water intake is generally an initial strategy for KSD prevention [24,25]. Throughout the past decade, the relevance of increased water intake to prevent KSD has been consistently confirmed [[26], [27], [28]].

Some of daily consumed beverages, such as coffee, can also exert diuresis. Similar to coffee, the diuretic effect of tea and alcohol has been also reported. A meta-analysis has reported that consumption of several beverages, such as coffee, tea, and alcohol, may be associated with a lower risk of KSD [29]. A recent population-based, prospective cohort study analyzing the data obtained from 439,072 participants from the UK Biobank has also shown that high fluid intake and consumption of coffee, tea, and alcohol can reduce hospitalization for the first stone episode [30].

From an in vitro stone formation study, it is not unexpected that CaOx crystallization and aggregation decrease after the urine is diluted by hydration in both normal subjects and stone formers [31]. Expression of a calcium stone modulatory protein, osteopontin (OPN), is also affected by the hydration status [32]. Note that OPN exhibits dual modulatory roles in kidney stone formation [33]. Although it promotes CaOx crystal adhesion on renal tubular cell surfaces [34] and self-aggregation [35], it inhibits crystal growth [36]. OPN expression significantly increases in renal tissue of dehydrated rats, whereas the high fluid intake reduces tissue expression of this protein [32]. Moreover, renal papillary density, which is elevated in stone formers, significantly declines in patients with increased fluid intake for 12 mo [37]. All these findings indicate that high fluid intake prevents KSD not only by increasing the rate of crystal elimination (through higher urinary flow rate) but also by affecting other stone formation steps, such as crystallization, crystal–cell adhesion, and crystal aggregation.

### Lifestyle and habit modifications

Obesity and overweight are also considered as risk factors for KSD [38,39]. Hence, weight loss to maintain the normal body mass index (BMI) is recommended by the EAU and UAA to reduce risk of KSD [10,12]. An in vivo study has also revealed that weight loss by food restriction and exercise can increase urinary citrate excretion and reduce risk of KSD [40]. Physical activity and exercise can reduce and protect several diseases and disorders. A large cohort study of almost 90,000 women has suggested that physical activity (regardless of its intensity) may prevent KSD, as evidenced by the association of physical activity with a lower risk of KSD in postmenopausal women [41]. A meta-analysis from 3 large prospective cohorts comprising >200,000 participants in total has reported the association of a lower risk of incident kidney stones with a higher level of physical activity in women [42]. However, there is no independent association found after multivariate adjustment [42]. Similarly, a later meta-analysis has also shown no association between physical activity and risk of KSD [43]. However, an analysis of questionnaire survey in patients with KSD from Southern China has revealed that physical activity is one of the protective factors for KSD [44]. An inverse correlation of physical activity and KSD prevalence is found in both genders. In addition, some protective factors, such as high urinary magnesium, are found in athletes [45]. On the contrary, several risk factors for kidney stone formation (such as dehydration, high urinary calcium, uric acid and sodium concentrations, and low urinary citrate concentration) are also found in these athletes [45]. From these data, the true benefit of physical activity and exercise on KSD remains controversial and needs further elucidations with adjustments on several variables or confounding factors. Another factor that should be also considered is hydration status during and after heavy physical activity and exercise, which can induce profound sweating and water loss.

The effects of working environment and climate change on risk of KSD have been also investigated [[46], [47], [48]]. Workers, who have been working in steel industry with high-temperature area, experience increased risk factors for kidney stone formation, such as hypocitraturia and low urine volume [49]. Thus, the KSD prevalence among them is higher than that of the general population [50]. In concordance, the civil construction workers, who often work in a high-temperature environment, experience the higher KSD prevalence than the general population [51]. From these data, the higher KSD prevalence related to the high temperature seems to be induced by profound sweating and dehydration. Hence, increasing fluid intake may be considered to reduce risk of KSD among these workers.

Although several studies have clearly shown the increased risk of several kidney diseases in cigarette smokers, it is surprising that the relevance of smoking in KSD is controversial. One of these studies has shown that cigarette smoking increases risk of KSD because the percentage of smokers in stone formers is higher than that in healthy individuals [52]. However, later studies have shown no significant association between cigarette smoking and KSD risk [53] and its recurrence [54]. A recent study in Iran found that the stone formers record a higher cigarette smoking habit than healthy subjects, but no association was found after adjusting with other factors [55]. However, a most recent study from Taiwan demonstrated that passive smoking in nonsmokers is associated with the higher KSD incidence than the unexposed nonsmokers [56]. These data suggest that secondhand smoke is the risk for kidney stone development [56]. Based on these references, avoidance of cigarette smoking and secondhand smoke should be recommended to prevent KSD, but stronger evidence is still required.

### Dietary management

Some dietary styles, such as Mediterranean diet pattern (defined as high consumption of plant-derived foods with monounsaturated to saturated fatty acids and low consumption of meats), are associated with a lower risk of KSD [[57], [58], [59]]. Several metabolic abnormalities, such as hypercalciuria, hyperoxaluria, hypocitraturia, hypomagnesuria, and hyperuricosuria, are the known risk factors for kidney stone formation. Therefore, dietary modifications may correct these metabolic abnormalities, lower KSD risk, and prevent new and recurrent kidney stones [[60], [61], [62], [63], [64], [65], [66]].

#### Calcium intake and vitamin D supplementation

As hypercalciuria is one of the major risk factors for calcium stone, daily calcium intake is an issue of concern. The AUA, EAU, CUA, and UAA consistently recommend that patients with calcium stone should consume dietary calcium at 1000–1200 mg/d [[9], [10], [11], [12]]. There is an evidence showing that calcium supplement increases risk of KSD [67]. On the contrary, a high dietary calcium reduces symptomatic KSD (owing to the binding of dietary calcium with dietary oxalate in the gut, thereby reducing gastrointestinal absorption of oxalate) [67,68]. There are several factors that lead to hypercalciuria, such as high renal acid load by diets [69], metabolic bone disease [70], and hyperparathyroidism [71]. Intestinal absorption of calcium can be enhanced by supplementary vitamin D. Consuming dietary calcium together with vitamin D has a synergistic promoting effect on kidney stone formation in rats [72]. Another in vivo study has confirmed that co-administration of calcium and vitamin D aggravates Randall plaque formation [73]. The retrospective study of calcium stone formers has revealed that vitamin D supplement increases urinary calcium excretion [74]. However, a meta-analysis has reported that long-term supplementation of vitamin D increases risk of hypercalciuria but does not increase risk of KSD [75]. Another prospective analysis of almost 200,000 participants has reported that the association between vitamin D intake and risk of KSD does not reach a statistically significant threshold [76]. Similarly, a randomized controlled trial has revealed that monthly vitamin D supplementation does not increase risk of KSD and serum calcium concentration [77]. Note that the subtype of the calcium stone should be considered in these cases with contradictory results. Therefore, risk factors and calcium and vitamin D concentrations of individuals should be also considered before deciding dietary calcium intake and vitamin D supplementation.

#### Sodium intake

High dietary sodium ingestion is accompanied by increased urinary calcium excretion [78,79] and CaOx crystal deposition in the kidney [80,81]. In pediatric patients, higher dietary sodium intake is also associated with calcium urolithiasis [82]. In support of this observation, an in vivo study performed in genetic hypercalciuric stone-forming rats has revealed that low dietary sodium chloride intake reduces urinary calcium excretion and kidney stone formation [83]. Similarly, a retrospective analysis has found that low-sodium diet causes a significant reduction of urinary calcium [84]. These data consistently indicate that the high sodium intake increases risk of kidney stone formation, whereas sodium restriction is promising to prevent KSD. According to the AUA guidelines, patients with calcium stone should limit dietary sodium intake to ≤100 mEq/d (2300 mg/d) [9]. Moreover, both UAA [12] and EAU [10] suggest restriction of sodium intake in patients with calcium stone to 2 g/d (3–5 g/d of NaCl).

#### Oxalate intake

Hyperoxaluria is another major risk of KSD. Exogenous oxalate is mainly from oxalate-rich foods, whereas endogenous oxalate is produced by liver and erythrocytes [85,86]. Hyperoxaluria can be classified as primary and secondary hyperoxaluria. Defects in liver enzymes involved in glyoxylate metabolism lead to oxalate overproduction and are the common cause of primary hyperoxaluria [85,86]. Hence, liver injury is associated with primary hyperoxaluria [87,88]. Secondary hyperoxaluria is caused by many factors, such as ingestion of high oxalate and oxalate precursor–rich foods and increased intestinal absorption of oxalate due to malabsorption syndrome [85,86]. The urinary oxalate concentration is mainly influenced by ingestion of dietary oxalate and its precursor [89]. An in vitro study has shown that high oxalate enhances the capability of renal epithelial cells to bind COM crystals [22]. Such enhancement of COM crystal-binding capability is mediated through the increased surface expression of α-enolase, one of the COM-binding proteins [22]. Therefore, restriction of oxalate-rich foods, such as spinach, soy products, nuts, almonds, potatoes (particularly skin part), beets, navy beans, raspberries, and dates [90], is one of the recommendations for CaOx kidney stone prevention in all guidelines [[9], [10], [11], [12],^91^]. Interestingly, the DASH-style diet has been shown to increase urinary oxalate, magnesium, and citrate excretion and decrease urinary CaOx supersaturation [62]. Therefore, this dietary style may be considered as an alternative to the low-oxalate diet. Urinary oxalate concentration also depends on gastrointestinal oxalate absorption, which can be influenced by dietary calcium and microbiota within the gastrointestinal tract (see the Probiotics Section below). Enteric hyperoxaluria caused by the increased intestinal (colon) absorption of oxalate owing to gastrointestinal malabsorption is common in patients after gastric bypass or bariatric surgery [92,93]. The nonabsorbed negatively charged fatty acids then bind with enteric calcium in small intestine, leading to reduced enteric CaOx precipitation and increased free oxalate to enter and be absorbed at the colon. Thus, gastric bypass/bariatric surgery is associated with the high risk of KSD [94]. The AUA has specified that patients with CaOx stones and hyperoxaluria should limit oxalate-rich foods but keep sufficient calcium intake (1000–1200 mg/d) (to allow enough calcium to bind oxalate in the gut before oxalate enters the colon) [9].

#### Vitamin C intake

Vitamin C or ascorbic acid in human body is mainly from dietary source, particularly, fresh fruits and vegetables. It can be metabolized and converted to oxalate, which is further excreted into the urine. Several studies have reported that excessive intake of vitamin C (mostly in the form of vitamin C supplement) increases risk of kidney stone formation. An in vivo study has revealed that vitamin C can increase urinary oxalate excretion in hydroxy-l-proline–induced hyperoxaluric rats [95]. By contrast, vitamin E can ameliorate the hyperoxaluric effects of both hydroxy-l-proline and vitamin C [95]. Vitamin E also reduces renal deposition of COM and COD crystals in hydroxy-l-proline–induced hyperoxaluric rats [95]. On the contrary, an in vitro study has shown antioxidative property of vitamin C with preventive effect against oxalate-induced oxidative stress and renal injury [96]. Moreover, another in vitro study has suggested that vitamin C inhibits struvite crystallization in the presence of *Pseudomonas aeruginosa* [97].

#### Animal versus plant proteins

The association of dietary protein ingestion and KSD risk depends on the type of those dietary proteins [98]. Ingestion of animal proteins, such as fish, beef, and chicken, is associated with a higher risk of KSD, especially calcium and uric acid stones [98,99]. Hence, the EAU and UUA guidelines specify that animal proteins should be limited to 0.8–1.0 g/kg body weight/d in patients with calcium stone and those with hyperuricosuria because the animal proteins are associated with low urinary pH and citrate concentration and high concentrations of urinary oxalate and uric acid [10,12]. Although without amount specification, the CUA also recommends a moderate consumption of animal proteins [11], whereas the AUA recommends limiting consumption of nondairy animal proteins in patients with calcium and uric acid stone [9]. The renal acid load by diet from animal-derived foods is also associated with calcium and citrate metabolism, leading to increased urinary calcium excretion and reduced urinary citrate excretion [69,100]. By contrast, plant protein intake shows the protective effects [99]. Therefore, increasing the ratio of plant to animal proteins in foods is recommended for KSD prevention.

#### Citrate intake

Another main risk factor for KSD is hypocitraturia. Hypocitraturia can be induced by several factors as mentioned earlier and by the use of some drugs, for example, carbonic anhydrase inhibitors (such as acetazolamide) for treatment of other diseases [101]. Patients with kidney stones are encouraged by the AUA, EAU, CUA, and UAA to increase consumption of fruits and vegetables to increase fibers and urinary citrate concentration, particularly in patients with hypocitraturia [9]. Citrus fruits are the main source of dietary citrate, and grapefruit and lemon juices provide the highest and second highest citrate concentrations, respectively, compared with other citrus fruit juices [102]. Consumption of orange soda, a citrate-rich beverage, can also increase urinary citrate excretion [103]. A recent study has suggested that lemonade consumption may provide a cost-effective option for reduction of recurrent calcium stone by increasing urine volume and citrate concentration and decreasing supersaturations of CaOx and CaP [104]. Dietary supplement by fresh lemon juice can also prevent the recurrence of CaOx stone [105]. In addition, lime powder inhibits COM crystal growth and prevents reactive oxygen species overproduction in COM crystal–induced tubular cell injury [106]. Clinical trial in healthy volunteers has revealed that lime powder enhances urinary citrate excretion, increases urine pH, and decreases urinary CaOx supersaturation [107]. Interestingly, there is no toxic effect of lime powder observed in in vitro cell culture, in vivo mice model, and healthy individuals, suggesting that lime powder should be safe for daily consumption [106]. Moreover, lime powder decreases 24-h urinary (total) protein excretion but increases urinary uromodulin concentration in stone formers when compared with placebo [108]. Note that uromodulin has dual modulatory effects on kidney stone formation [33]. Under some particular circumstances, this protein can promote crystal aggregation [109]. However, several in vitro and in vivo studies have reported that uromodulin serves as a potent inhibitor of calcium stones [110,111]. Therefore, lime powder prevents KSD by increasing urinary concentrations of citrate and uromodulin, both of which are the potent inhibitors of calcium stone formation.

### Natural bioactive compounds

During the past decade, the beneficial roles of dietary plants in KSD [[112], [113], [114], [115], [116]] and the antilithogenic activities of various plant extracts [[117], [118], [119], [120], [121], [122], [123], [124], [125]] and bioactive compounds [[126], [127], [128], [129], [130]] have been widely investigated. Recent reviews have summarized evidence in dietary/medicinal plants and their bioactive compounds for kidney stone prevention and management [131,132]. Herein, we select to discuss some of these antilithogenic bioactive compounds with recent studies on their molecular mechanisms.

#### Caffeine

Caffeine is a natural xanthine alkaloid and a major bioactive constituent in coffee beans. It is quite clear that caffeine consumption exerts the diuretic effect. Nevertheless, the association between caffeine consumption and KSD prevalence was previously controversial. A study on 39 calcium stone formers and 39 control subjects has reported that caffeine consumption increases CaOx precipitation index, thereby increasing risk of KSD [133]. A recent study has also shown that caffeine consumption is associated with a higher risk of recurrent KSD [134]. On the contrary, ≥3 large meta-analyses have consistently reported the antithetical association between the consumption of caffeinated beverages and KSD risk [29,112,135]. In other words, caffeine reduces risk of KSD. In concordance, a recent prospective cohort study has confirmed the declined risk of KSD by coffee consumption [30]. In addition, an in vitro study has supported the protective effects of caffeine against kidney stone formation [136]. The results indicate that caffeine has an antilithogenic effect by reducing COM-binding ability of renal epithelial cells [136]. Such a protective effect is mediated by the translocation of annexin A1, which is one of the COM-binding proteins, from apical surfaces to cytosolic compartment of renal epithelial cells derived from the distal nephron [136].

#### Epigallocatechin gallate

Epigallocatechin gallate (EGCG) is a major bioactive constituent found mostly in green tea. Although tea is known as a main source for oxalate, green tea consumption is inversely associated with risk of KSD [114]. Such negative correlation is more predominant in males than that in females [114]. Green tea supplement increases superoxide dismutase (antioxidant) activity, decreases urinary oxalate excretion, and reduces CaOx renal deposition in EG-induced kidney stone rats [137]. Another in vivo evidence has confirmed the protective effect of green tea, revealing that green tea decreases urinary oxalate excretion and renal deposition of CaOx crystals in sodium oxalate–administrated rats [138]. In addition, EGCG also prevents oxalate-induced renal cell damage and reactive oxygen species overproduction [138]. To address the mechanisms for which EGCG inhibits kidney stone formation, an *in vitro* study has reported that EGCG inhibits the oxalate-induced membrane translocation of α-enolase, another COM-binding protein, resulting in the reduction of COM-binding ability of renal epithelial cells [139].

#### Diosmin

Diosmin is a bioflavonoid found in several plants and fruits, especially citrus fruits. The antilithogenic effect of diosmin has been reported to protect CaOx deposition in the kidney of EG-induced nephrolithiatic rats [140]. Diosmin also reduces degenerative changes in the renal cortex and medulla [140]. A more recent in vitro study has systematically examined dual modulatory effects of diosmin on CaOx crystals [141]. During crystallization, diosmin increases CaOx crystal number and mass. On the contrary, it decreases CaOx crystal size and growth. For other processes, diosmin enhances CaOx self-aggregation and extracellular matrix invasion but reduces CaOx–cell interactions [141].

### Medications

Medications may be required in some individuals who are at a high risk of new or recurrent KSD. Many types of medicine are well known and widely used for prevention of the stone recurrence, such as diuretics, potassium citrate, potassium bicarbonate, sodium bicarbonate, allopurinol, tiopronin, acetohydroxamic acid, and sodium thiosulfate [10,[142], [143], [144], [145], [146], [147], [148], [149], [150]]. Drug-induced diuresis by using diuretics, such as thiazides, is recommended to prevent recurrent stones and to reduce urinary calcium excretion [151,152]. A consensus has been made by different guidelines to recommend the use of thiazides in recurrent calcium stone formers and those with hypercalciuria [153]. Type and dosage of these thiazides slightly vary: hydrochlorothiazide 25 mg twice daily (AUA, EAU, and CUA) or 50 mg once daily (AUA, EAU, CUA, and UAA); chlorthalidone 25 mg once daily (AUA, EAU, CUA and UAA) or 50 mg once daily (CUA); and indapamide 1.25 mg once daily (CUA) or 2.5 mg once daily (AUA, EAU and UAA) [[9], [10], [11], [12]]. A retrospective cohort study in elders has shown similar protective effects of thiazides against KSD at low and high doses [151]. However, long-term use of thiazides for recurrent KSD is not recommended because of its potential adverse effects [152]. If their long-term use is unavoidable, potassium supplementation (either potassium citrate or chloride) should be considered because hypokalemia is common with these agents [11,12].

Another commonly used drug for the prevention of kidney stone recurrence is alkaline citrate, particularly potassium citrate, which is recommended by the AUA, EAU, CUA, and UAA to alkalinize urine in patients with recurrent calcium stone, hypocitraturia, uric acid stone, and cystine stone [[9], [10], [11], [12]]. In addition to urinary alkalinization, an in vivo study on genetic hypercalciuric stone-forming rats has found that administration of potassium citrate leads to increased urinary citrate and decreased urinary calcium concentrations [154]. It is possible that citrate can bind calcium in the gastrointestinal tract, thereby reducing intestinal calcium absorption [154]. Citrate can also bind urinary calcium, leading to a decline of urinary calcium supersaturation [154]. A recent study has consistently shown that oral consumption of potassium citrate reduces urinary calcium excretion in CaOx stone formers with hypercalciuria [155]. Another recent study has shown that thiazides (particularly, chlorthalidone) is more effective in reducing CaP stone than potassium citrate [156]. Combination of these 2 drugs (chlorthalidone and potassium citrate) is better than each of them alone to prevent CaOx stone in hypercalciuric rats [157]. In addition, sodium and potassium bicarbonates are commonly used to correct metabolic acidosis (one of the inducers of hypocitraturia) [150,158,159]. Long-term intake of potassium citrate may lead to some adverse effects such as abdominal pain and other gastrointestinal symptoms [160]. Owing to these side effects and problem in affordability, alternative alkalinizing agents can be used for kidney stone prevention [161]. Although the alternative, over-the-counter, nonprescription oral alkalinizing agents significantly save cost, their citrate and alkali equivalents are reduced [162]. Further studies are required to address their cost-effectiveness compared with potassium citrate. The dosage of potassium citrate varies (3–10 mmol 2 or 3 times daily as recommended by the EAU and 30–60 mEq daily in 2 or 3 divided doses as documented by the CUA). More importantly, the goals of urinary pH are >6.0–6.5 (AUA, CUA, and UUA) for uric acid stone formers and >7.0–7.5 (AUA, EAU, CUA, and UUA) for cystine stone formers [[9], [10], [11], [12]].

Allopurinol is another medication for CaOx stone formers recommended by the AUA to prevent the stone recurrence in those with hyperuricosuria (regardless of the serum uric acid concentration) and normocalciuria [9]. However, the CUA recommends using allopurinol only in patients with calcium stones and hyperuricemia to prevent the stone recurrence but does not recommend its use in those with normouricemia [11]. Finally, the aforementioned drugs are also applicable for prevention of uric acid stone [142,163,164]. However, their adverse effects should be periodically checked.

### Bacterial eradication

UTIs by urease-producing bacteria are associated with struvite stone formation. Nevertheless, the etiologic effects of UTIs on other kidney stone types have been also evidenced [165]. Bacterial cultures of urine from stone formers and stone matrices (both nidus and periphery) have found *Escherichia coli* as the most common organism in all these types of clinical samples, implicating that *E. coli* may cause kidney stone formation [165]. Interestingly, only intact viable bacteria can promote CaOx crystal growth and aggregation, whereas dead intact and fragmented *E. coli* have no promoting effects [166]. A later study has reported that outer membrane vesicles (OMVs) derived from *E. coli* isolated from the urine of stone formers promote CaOx crystallization, crystal growth, and aggregation [167]. The promoting activities of OMVs from *E. coli* are mediated by elongation factor (EF)-Tu on the OMVs surface, and neutralization using specific antibody against EF-Tu markedly reduces such promoting activities [167]. Interestingly, EF-Tu is more abundant in *E. coli* isolated from the urine of stone formers compared with *E. coli* isolated from the urine of non–stone formers with UTIs [167]. In addition to OMVs, a recent study has reported that flagella derived from viable *E. coli* also promote CaOx crystallization, crystal growth, and aggregation [168]. In addition to *E. coli*, other bacteria, such as *Klebsiella pneumoniae*, *Pseudomonas aeruginosa*, and *Staphylococcus aureus*, are also found in stone nidus and cortex and the urine from stone formers [165]. These bacteria also exert promoting activities on CaOx growth and aggregation [166].

Conventionally, increased water intake is one of the strategies for preventing UTIs. Therefore, increased water intake and diuresis can lower risk of KSD in many ways. Removal of the infection sources (for example, stone removal) should be considered for bacterial eradication, particularly in patients with recurrent UTIs [169]. Additionally, the use of antibiotics based on bacterial culture from the urine and/or stone matrices may be able to prevent recurrent stone formation. However, bacterial eradication by antibiotics should be carefully considered because multidrug resistance has been already detected in bacteria isolated from the urine and stone matrices [165]. Some classes of antibiotics are associated with the increased risk of KSD or crystal-induced nephropathy [170]. The use of antibiotics for >2 mo in early adulthood and middle age is also associated with a higher risk of KSD in later life [171]. Moreover, a recent study has found that the use of antibiotics may suppress *Oxalobacter formigenes* in the gut microbiome [172]. Therefore, bacterial eradication by using antibiotics can be performed but with serious cautions and close monitoring.

### Probiotics

In contrast to uropathogenic bacteria, increasing evidence has shown that some probiotics, particularly oxalate-degrading bacteria (such as *Oxalobacter* spp., *Lactobacillus* spp., and *Bifidobacterium* spp. [[173], [174], [175], [176], [177], [178], [179], [180]]), have protective roles against KSD. *O. formigenes* is an anaerobic and oxalate-degrading bacterium. This type of probiotics normally resides within the intestinal tract. A recent study has revealed the relative less abundance of *O. formigenes* in the intestinal tracts of stone formers compared with healthy subjects [181]. Interestingly, the abundance of *O. formigenes* inversely correlates with urinary oxalate concentration [181]. Note that oxalate homeostasis in the gastrointestinal tract is due to the collaborative action between *O. formigenes* and numerous other microbiota, not *O. formigenes* alone [182]. In addition to *O. formigenes*, other probiotics, such as *Lactobacillus* spp. and *Bifidobacterium* spp., are also able to degrade intestinal oxalate and reduce urinary oxalate excretion [183,184]. Another recent study performed in *Drosophila melanogaster* model of urolithiasis has reported that *Bacillus subtilis* can reduce CaOx stone formation [185]. In addition, a more recent study has found that the diversity and abundance of gut microbiota largely differ under different dietary patterns/styles [186]. Therefore, the efficacy of gut probiotics to prevent KSD can be affected by diets.

## Summary

Prevention of the new and recurrent stones is the ultimate goal for management of KSD to reduce its physical, mental, and economic burdens. Currently, there are several protective strategies for kidney stone prevention recommended by the AUA, EAU, CUA, and UAA. Most of these guidelines are consistent, but some details differ. The choice of such protective strategies depends on etiology and type of kidney stones and cost-effectiveness of the method. A brief summary of such protective strategies based on the recent evidence is as follows:

(1) Increase fluid intake and diuresis
  - As dehydration is one of the major risks of KSD, this method seems to be the easiest and most cost-effective strategy. The fluid intake at 2.5–3.0 L/d with urine output >2.0–2.5 L/d is highly recommended.
(2) Lifestyle and habit modifications
  - Maintaining or reducing the BMI to the normal range is recommended.
  - Although health benefits of physical activity and exercise are well-recognized, their benefits for kidney stone prevention remain to be elucidated. In addition, it is possible that dehydration during and after heavy physical activity and exercise is one of the major determinants. Therefore, fluid compensation during and after heavy physical activity and exercise may be the solution.
  - Working under high temperature may leads to dehydration. Therefore, fluid compensation is recommended.
  - Avoidance of cigarette smoking and secondhand smoke. However, a stronger evidence is still required.
(3) Dietary management
  - Patients with calcium stone should consume sufficient dietary calcium at 1000–1200 mg/d.
  - Limiting sodium intake in patients with calcium stones to 2 g/d (3–5 g/d of NaCl).
  - Limiting oxalate-rich foods, such as spinach, soy products, nuts, almonds, potatoes (particularly skin part), beets, navy beans, raspberries, and dates, in patients with CaOx kidney stones.
  - Avoidance of vitamin C and vitamin D supplements. Because there are pros and cons of using these vitamin supplements, further elucidations are still required.
  - Limiting the intake of animal proteins (0.8–1.0 g/kg body weight/d) but increasing plant proteins in patients with calcium and uric acid stones and those with hyperuricosuria.
  - Increase proportion of citrus fruits (particularly grapefruit, lemon, and lime) in daily consumed foods and juices. Lime powder supplement is another choice to increase urinary citrate and uromodulin excretion to prevent calcium stone formation.
(4) Natural bioactive compounds
  - Natural bioactive compounds should be used with cautions. Several lines of earlier evidence were controversial, and further investigations in larger cohorts are required.
  - Caffeine consumption may be recommended. Although earlier evidence was controversial, several lines of recent (and more solid) evidence in prospective cohort and in vitro molecular mechanisms studies have consistently demonstrated the protective effects of caffeine against KSD.
  - Recent preclinical evidence has shown beneficial effects of EGCG.
  - The protective role for diosmin remains controversial. It seems that diosmin exerts dual modulatory activities on CaOx crystals. Therefore, additional and stronger evidence is required.
(5) Medications
  - The use of thiazides is recommended for recurrent calcium stone formers and those with hypercalciuria. The recommended thiazides include hydrochlorothiazide 25 mg twice daily or 50 mg once daily, chlorthalidone 25 or 50 mg once daily, and indapamide 1.25 or 2.5 mg once daily.
  - Alkaline citrate, particularly potassium citrate, is recommended to alkalinize urine in patients with recurrent calcium stones, hypocitraturia, uric acid stones, and cystine stones.
  - Although the alternative, over-the-counter, nonprescription oral alkalinizing agents significantly save costs, their citrate and alkali equivalents are reduced. Further studies are required to address their cost-effectiveness compared with potassium citrate.
  - The goals of urinary pH are >6.0–6.5 for uric acid stone formers and >7.0–7.5 for cystine stone formers.
  - Allopurinol is recommended for CaOx stone formers with hyperuricosuria and normocalciuria. It seems to be effective for those with hyperuricemia but not for those with normouricemia.
  - Potential side effects should be periodically monitored after a long-term use of medications.
(6) Bacterial eradication
  - Removal of the infection sources (for example, stone removal) should be considered for bacterial eradication, particularly in cases with recurrent UTIs.
  - The use of antibiotics may be able to prevent recurrent stone formation.
  - However, multidrug resistance has been already detected in bacteria isolated from the urine and stone matrices. The long-term use of antibiotics can also suppress gut microbiota, particularly *O. formigenes.* Therefore, their use must be carefully considered and requires monitoring.
(7) Probiotics
  - Supplementation of probiotics, such as *O. formigenes*, *Lactobacillus* spp., *Bifidobacterium* spp., and *B. subtilis*, to reduce intestinal oxalate absorption seems to be beneficial for kidney stone prevention. However, large prospective cohorts are required to strengthen its beneficial roles.

Finally, as summarized, several data sets from research activities during the past 10 y have pointed out that there are some new strategies for kidney stone prevention that worth for further elucidations in larger prospective cohorts to be considered by several various guidelines in the future.

## Funding

This work is supported by National Research Council of Thailand (NRCT): High-Potential Research Team Grant Program (N42A660625).

## Author disclosures

PP and VT, no conflicts of interest.

## Data availability statement

Data sharing does not apply to this review article as no new data were generated.
